# Suppression of collapse for two-dimensional Airy beam in nonlocal nonlinear media

**DOI:** 10.1038/s41598-017-04095-9

**Published:** 2017-06-23

**Authors:** Qian Kong, Ning Wei, Cuizhi Fan, Jielong Shi, Ming Shen

**Affiliations:** Department of Physics, Shanghai University, 99 Shangda Road, Shanghai, 200444 P. R. China

## Abstract

Dynamics and collapse of two-dimensional Airy beams are investigated numerically in nonlocal nonlinear media with split step Fourier transform method. In particular, the stability and self-healing properties of the Airy beams depend crucially on the location and topological charge of the vortex when the beams carry angular momentum. The propagation of abruptly autofocusing Airy beams is also demonstrated in local and nonlocal media. In strongly self-focusing regime, with the help of nonlocality, stationary propagation of two-dimensional Airy beams can be obtained, which always collapse in local nonlinear media.

## Introduction

Self-accelerating Airy beams^1, 2^ have been a hot topic^3–5^ during the past decade, which shown that Airy beams have potential applications in different physical settings, such as particle clearing^6^, surface plasmon^7^, and generation of electron Airy beam^8^, etc. On the other hand, the dynamics of Airy beam propagating in nonlinear media^9–13^ have also drawn considerable attention, e.g., nonlinear generation of Airy beams^14^, spatial Airy solitons^15, 16^, spatiotemporal Airy light bullets^17, 18^, as well as interactions and manipulation of Airy beams^19–23^. Furthermore, another interesting phenomena of collapse may happen when the effect of nonlinearity impact on an two-dimensional Airy beam^24–26^. In particular, Airy beams with higher powers in the main lobe were reshaped into a multifilamentary (collapse into particles) pattern induced by strong nonlinearity, which will affect the acceleration of the main Airy lobes^25^. When a vortex Airy beam propagating in a nonlinear media, the locations of collapse can be controlled by the initial power, vortex order, and modulation parameters^26^. In three dimension, under the action of strong self-focusing Kerr nonlinearity, the rapid collapse of three-dimensional Airy-vortex wave packets imposes a severe limitation on the propagation of the beam^27^.

In general, the collapse effect represents a strong mechanism for energy localization^28, 29^, which is a fundamental physical phenomenon well known in different physical subjects, such as plasma^30^, optics^31^, Bose-Einstein condensates^32^, and black hole of stars^33^. Different suggestions have been made on how to arrest the collapse, e.g., saturation of the nonlinearity^29^, alternating focusing-defocusing layers^34^, and optical gain^35^. Besides, recent studies have demonstrated that the application of nonlocal response (nonlinearity) is an effective way to prevent the catastrophic collapse of high-dimensional waves in nonlinear media^36^.

In optics, nonlocality means that the light-induced refractive index change of a material at a particular location is determined by the light intensity in a certain neighborhood of this location^37^. Nonlocal nonlinearity exists in nematic liquid crystals^38^ and thermal media^39^. For the Airy beams, nonlocal nonlinear affect deeply the propagation dynamics of self-accelerating Airy beams^40–42^ and their interactions^42–44^. What’s more, one of the most important properties of nonlocal nonlinearity is that it eliminates collapse in all physical dimensions for arbitrary shapes of the nonlocal response, as long as the response function is symmetric and has a positive definite Fourier spectrum^36^.

In this paper, we investigate numerically dynamics and collapse of two-dimensional Airy beam in nonlocal nonlinear media. The stability and self-healing properties of the beams depend crucially on the location and topological charge of the vortex when the Airy beam carrying angular momentum. We also demonstrate the propagation of abruptly autofocusing Airy beam in detail. In strongly self-focusing regime, with the help of nonlocality, we obtain the stationary propagation of two-dimensional Airy beams, which always collapse in local nonlinear media.

## Results

### Dynamics of two-dimensional conventional Airy beam in nonlocal nonlinear media

**Figure 1:**
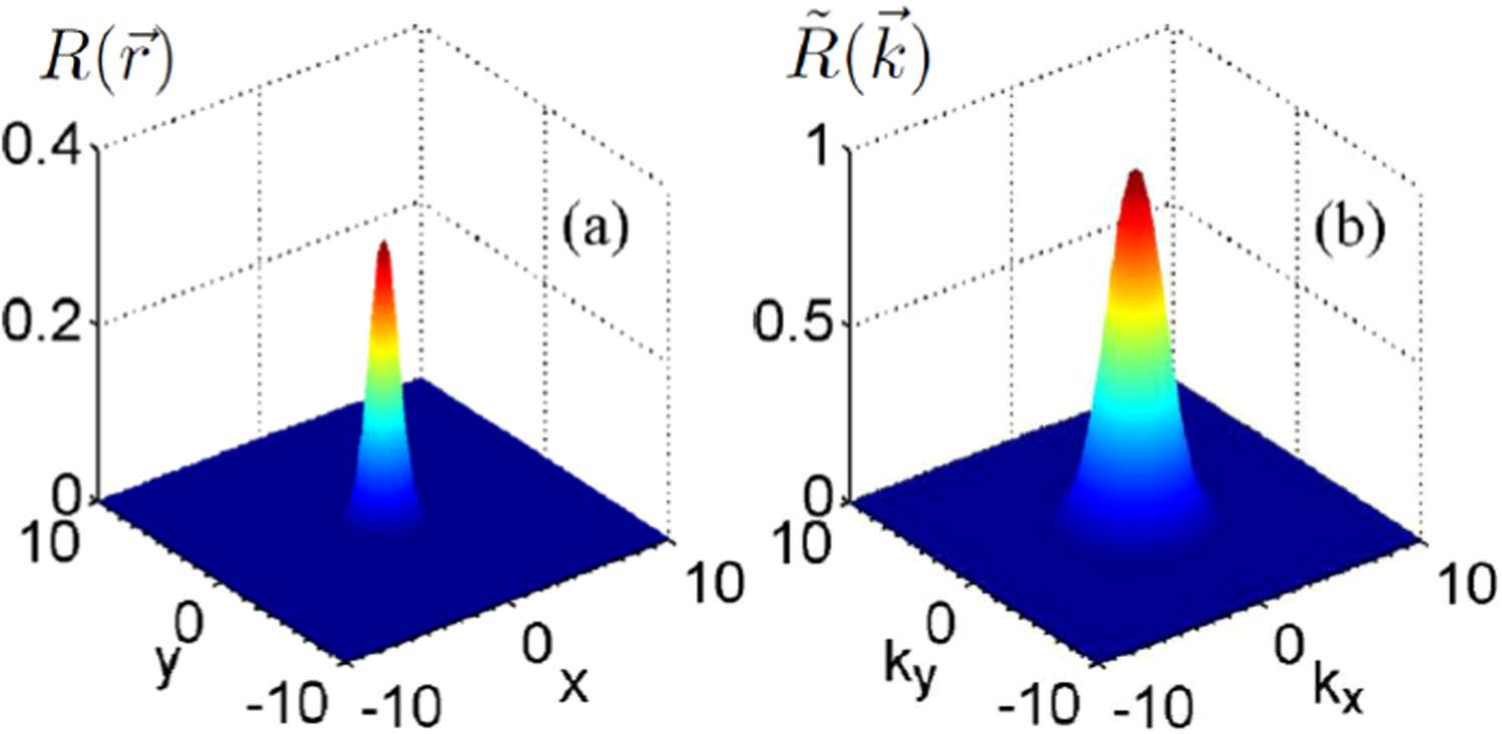
The profile of the Gaussian nonlocal response function (**a**) and its Fourier transform in (**b**).

In this section, we study the dynamics of conventional two-dimensional Airy beam in nonlocal nonlinear media by direct numerical simulations with split-step Fourier transform method^42^. The profile of the nonlocal response function and its Fourier transform are shown in Fig. 1(a,b), respectively. We also assume the Airy beam is in the following form carrying optical vortex characterized by the topological charge *m* ^45, 46^

1 $$\psi (x,y,z=0)=A\cdot Ai(x/w)Ai(y/w)\exp [a(x/w+y/w)]\times {[(x+{x}_{0})+i(y+{y}_{0})]}^{m},$$

where *A* is the amplitude of the Airy beam, *Ai*(*x*/*w*) and *Ai*(*y*/*w*) denote the Airy functions, *w* is the beam width of the Airy beam, *x*_0_ and *y*_0_ denote the dislocation of the optical vortex from the origin along the x and y axes^45, 46^, and *a* > 0 is the the decaying factor to ensure containment of the infinite Airy tail and the finite power of the Airy beam^1^. For simplicity, we set *w* = 0.5 and *a* = 0.05 throughout this paper.

**Figure 2:**
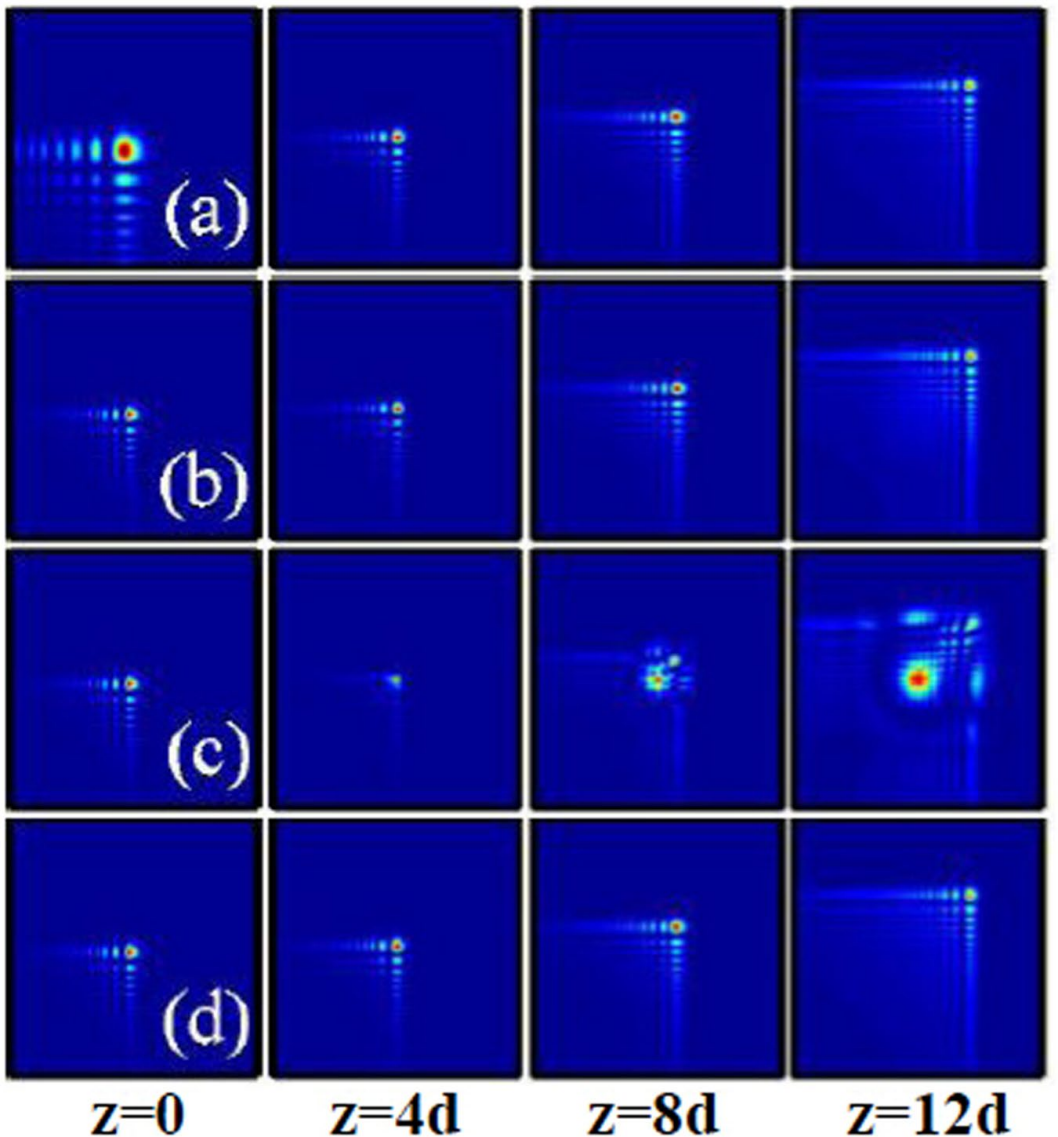
The intensity distributions of two-dimensional fundamental Airy beam at different propagation distances in (**a**) free space, (**b**,**c**) local self-focusing nonlinear media (*σ* = 0), and (**d**) nonlocal nonlinear media, respectively. The initial parameters are *m* = 0, *x*_0_ = *y*_0_ = 0, *A* = 5 (**a**,**b**), and *A* = 10 (**c**,**d**). The degree of nonlocality of Fig. 2(d) is *σ* = 2.

Firstly, the dynamics of two-dimensional fundamental Airy beam (the topological charge is *m* = 0) with lower power (smaller amplitude) propagating in linear media (free space) at different propagation distances *z* are displayed in Fig. 2(a). Here the propagation distances *z* is scaled by the diffraction length (Rayleigh range) *d* = *w* ^2^/2. For comparison, we also show in Fig. 2(b) the intensity distributions of such beam in local self-focusing nonlinear media (*σ* = 0). From Fig. 2(a,b), it is obviously that the Airy beams self accelerate and travel along the identical accelerating trajectory during propagation in local nonlinear medium and free space^24^. However, the field distributions of the Airy beam during propagation in local nonlinear medium are different from that in free space. When the beam propagates in a local nonlinear medium, the intensity distribution of the central parts of the Airy beam becomes more intensive and the intensity distribution of the sides of the Airy beam becomes weaker than that of the beam during propagation in free space^24^, as shown in Fig. 2(a,b).

It is interesting that with further increasing initial amplitude (power) of the Airy beam in local nonlinear media, the intensity at the central parts of the major lobe dominates and eventually leads to a catastrophic collapse of the Airy beam, while the beam width still remains constant, as displayed in Fig. 2(c). This is because the linear effects, including diffraction, self-accelerating, and self-healing, can not balance the effect of strong self-focusing. In the regime of strongly nonlinear media, the nonlinearity may show manifestations of linear dynamics of the Airy beam, where part of a linearly propagating Airy beam profile was obscured and observed to be reconstructed after a few propagation distances^25^. Thus the Airy beam eventually undergoes a collapse into a multi-filaments pattern.

The collapse behavior of the Airy beam can be effectively prevented with the help of nonlocality, as shown in Fig. 2(d). It has been shown that nonlocality eliminates collapse in all physical dimensions for arbitrary shapes of the nonlocal response, as long as the response function is symmetric and has a positive definite Fourier spectrum^36^. In this paper, the Fourier transform of a Gaussian response function indeed has a positive definite Fourier spectrum, as shown in Fig. 1(b). Thus the nonlocality induces an effective attractive potential, which can completely suppress the collapse of the Airy beam, leading to the stable propagation of Airy beam even when their power is large enough. Besides the suppression of the collapse, we also numerically study the effect of nonlocality impacts on the propagation of Airy beams (not shown). The dynamics of Airy beams in weakly nonlocal media are similar with that in local Kerr media^47^. However, in the regime of strong nonlocality, the change of nonlinear refractive index of the media trends to linear^48^, and the dynamics of Airy beams are similar with that in free space.

**Figure 3:**
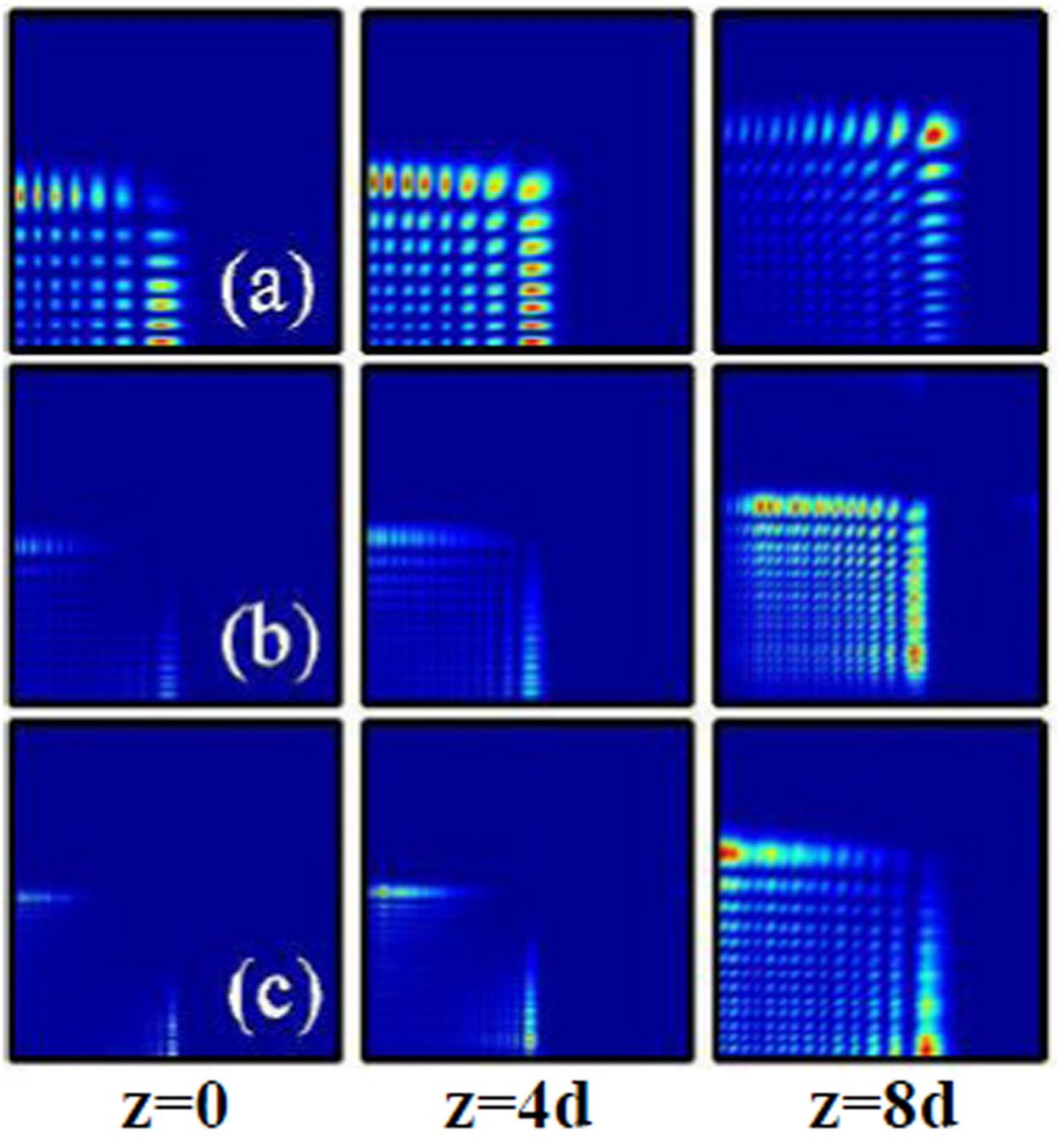
The intensity distributions of a vortex Airy beam at different propagation distances in local self-focusing nonlinear media (*σ* = 0). The initial parameters are *x*_0_ = *y*_0_ = 0, *A* = 2 (**a**), *A* = 0.2 (**b**), and *A* = 0.01 (**c**). The topological charges are *m* = 1 (**a**), *m* = 2 (**b**), and *m* = 3 (**c**), respectively.

When *m* ≠ 0, Eq. (7) represents an Airy beam carrying an optical vortex, which carries an orbital angular momentum^45, 46^. In Fig. 3(a–c), we show the propagation dynamics of the lower powered vortex Airy beam in local nonlinear media with the topological charge *m* = 1, *m* = 2, and *m* = 3, respectively. We also assume that the vortex centers are located at the position of the main lobe of the Airy beam, i.e., *x*_0_ = 0 and *y*_0_ = 0. We also numerically check that these dynamics of the vortex Airy beams are almost similar with that in free space (not shown), for that, in the weakly nonlinear regime, the linear dynamics prevails and the parabolic trajectory of the Airy peak is unperturbed^25^. Many works have investigated the transverse acceleration velocity or speed of these vortex Airy beam^45^, however, we mainly focus on the self-healing properties^49–51^ of the vortex Airy beam in this paper.

From Fig. (3), the self-healing of the vortex Airy beam is apparent during the propagation. The main lobe is reborn at the corner again and persists undistorted. The vortex Airy beam evolves into a new fundamental-like Airy beam after the reconstruction during the self-healing process. In particular, the self-healing properties of the vortex Airy beam depend crucially on the topological charge *m*. To ensure that the vortex Airy beams could propagate stably and the self-healing could happen, the amplitudes (power) of the beams should be smaller (larger) for larger (smaller) topological charges *m*. Furthermore, for vortex Airy beam with smaller topological charge *m* = 1, the beam is very easy to realize self-healing after few propagation distances, as shown in Fig. 3(a). While for the vortex Airy beam with larger topological charge, it is hard to realize self-healing at the same propagation distance, as shown in Fig. 3(b,c). It always requires larger distance for vortex Airy beam carrying larger topological charge to realize self-healing (not shown) due to the fact that the stability of optical vortex will become worse when its topological charge *m* increases.

**Figure 4:**
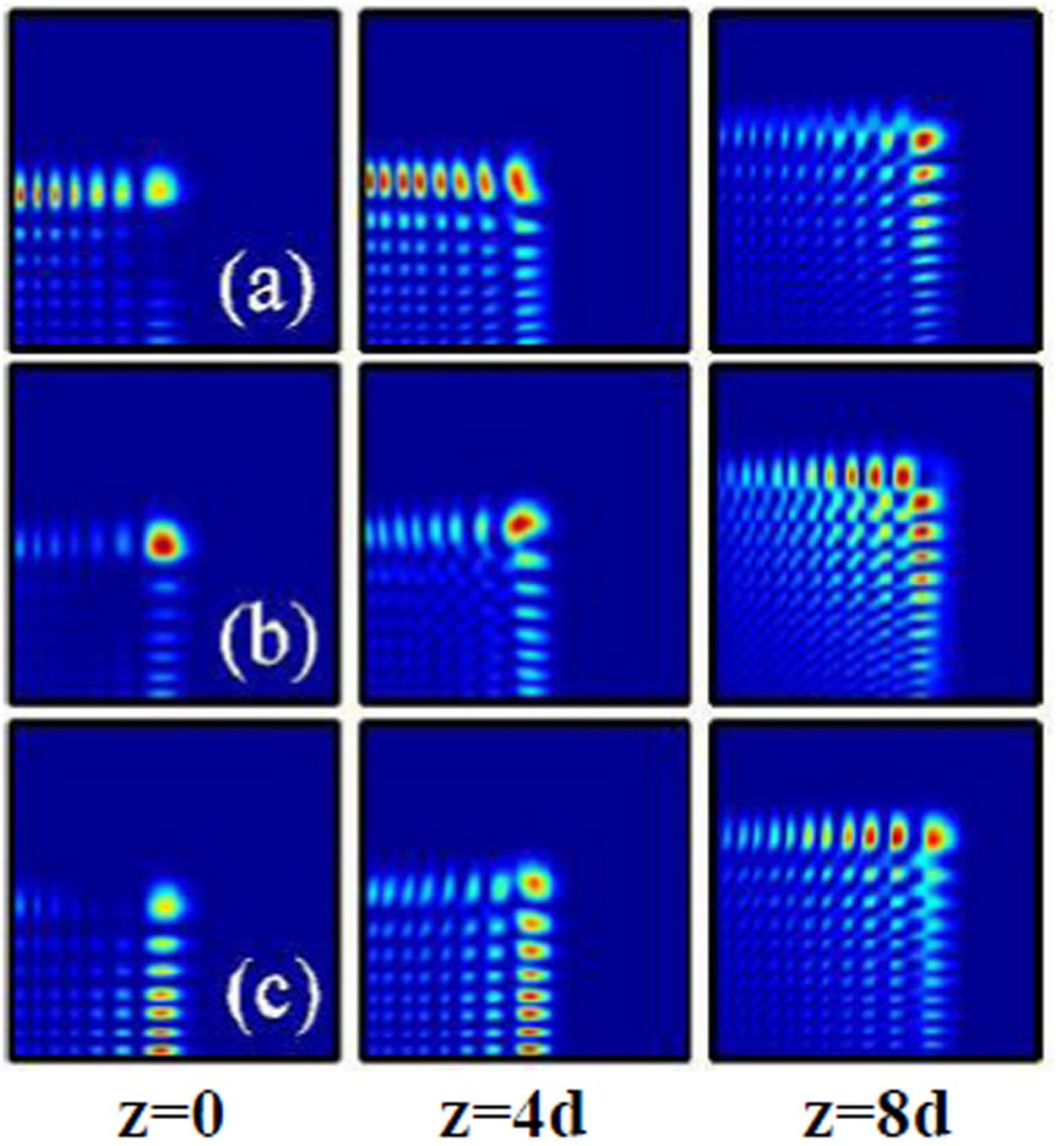
The intensity distributions of a vortex Airy beam at different propagation distances in local self-focusing nonlinear media (*σ* = 0). The amplitude and the topological charge of the beam are *A* = 2 and *m* = 1. The location of the vortex are (**a**)*x*_0_ = 0, *y*_0_ = 2, (**b**)*x*_0_ = 2, *y*_0_ = 2, and (**c**)*x*_0_ = 2, *y*_0_ = 0, respectively.

We also find that the self-healing properties of the vortex Airy beam depend on the location of the optical vortex center. In Fig. (4), we show the properties of self-healing of lowest order vortex Airy beam (the topological charge *m* = 1, higher order topological charge can be treated in similar way) with different vortex positions *x*_0_ and *y*_0_ in local nonlinear media. After self-healing process, the vortex Airy beam turns into fundamental Airy beam, however, their intensity distributions are different with different *x*_0_ and *y*_0_. When *x*_0_ = 0 and *y*_0_ = 2, the optical vortex is located at minus *y* axis, the intensity along *x* axis is obviously stronger than that along *y* axis. At the propagation distance *z* = 8*d*, the beam realizes self-healing. Whereas, the intensity along *y* axis is stronger than that along *x* axis after self-healing, as shown in Fig. 4(a).

This phenomena can be explained by studying the internal transverse power flow of the vortex beam^49^. The power flows from the lobes along *x* axis towards the lobes along *y* axis in order to facilitate self-healing. The similar result with the parameters *x*_0_ = 2 and *y*_0_ = 0 is shown in Fig. 4(c). Another strange and interesting result happens when *x*_0_ = 2 and *y*_0_ = 2, as shown in Fig. 4(b), the main lobe disappears instead of two secondary lobes after self-healing. On the contrary, the intensity distributions along both *x* axis and *y* axis increase accordingly. This indicates that when the vortex is located at the internal lobes, the power of the main lobe will flow towards the vortex along the 45° axis during self-healing process^49^.

**Figure 5:**
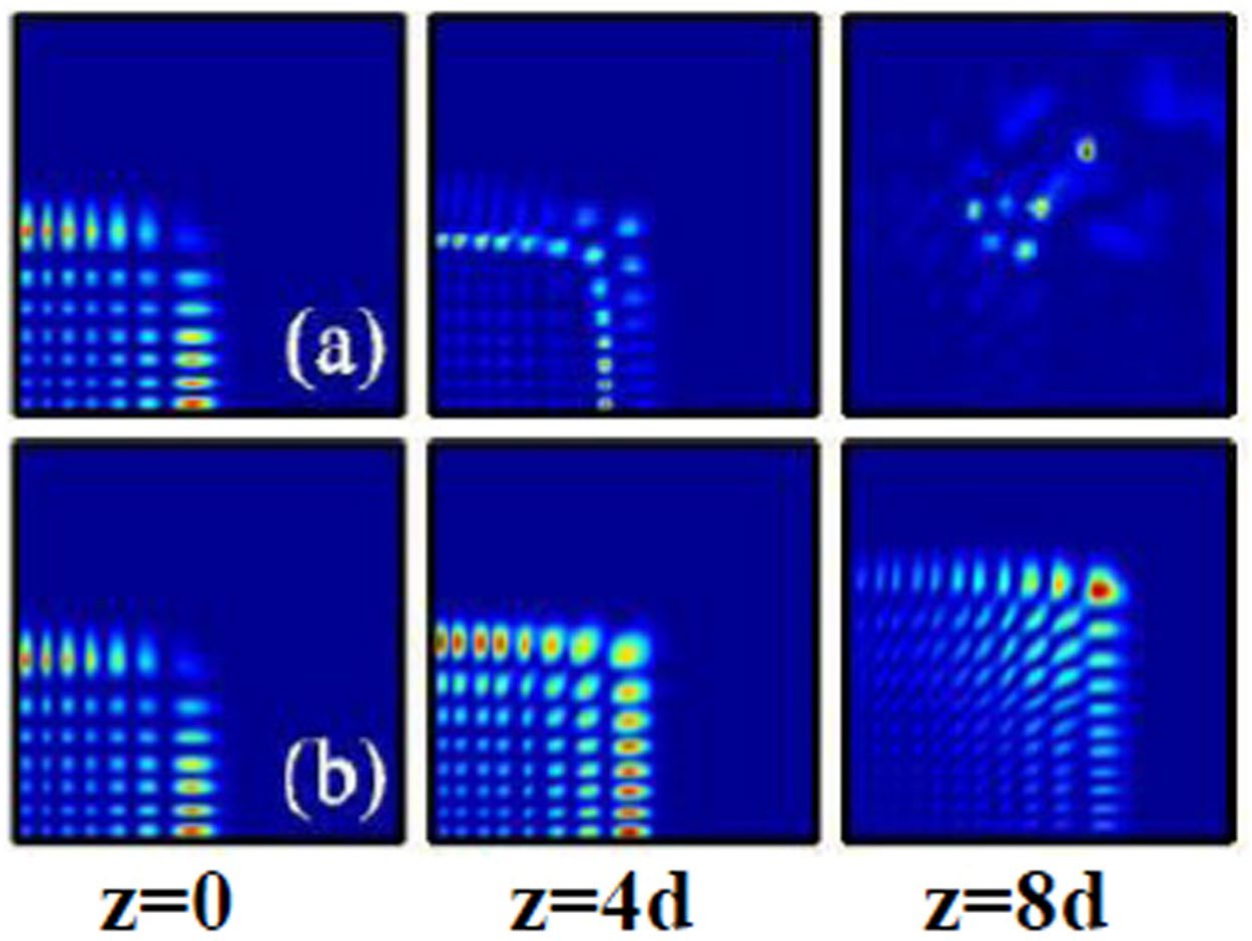
The intensity distributions of a vortex Airy beam at different propagation distances in (**a**) local nonlinear (*σ* = 0) and (**b**) nonlocal nonlinear media, respectively. The initial parameters are *m* = 1, *x*_0_ = *y*_0_ = 0, and *A* = 8, respectively. The degree of nonlocality of Fig. 5(b) is *σ* = 1.

With increasing the amplitude of the vortex Airy beam, the beam will also suffer from catastrophic collapse due to strong self-focusing effect, as displayed in Fig. 5(a). Here we only consider the case of *m* = 1 and *x*_0_ = *y*_0_ = 0. It should be emphasized that the detailed collapse dynamics of the vortex Airy beam in local nonlinear media have been extensively demonstrated by R. Chen *et al*.^26^, which shown the possibility of controlling and manipulating the collapse, especially the precise position of collapse, by purposely choosing appropriate initial power and topological charge of the vortex Airy beam. In nonlocal nonlinear media, as shown in Fig. 5(b), we can obtain the stationary propagation and the self-healing of the vortex Airy beam with the help of nonlocality, which is always collapse in local media.

### Dynamics of two-dimensional abruptly autofocusing Airy beam in nonlocal media

In this section, we consider the propagation dynamics of radially symmetric waves, i.e., abruptly autofocusing waves^52, 53^, propagating in nonlocal media. This new families of Airy beams have some unique properties, such as they can autofocus by following a parabolic trajectory toward their focus, and suddenly increases by orders of magnitude right before its focal point^52^. It has been shown that the new class of Airy beams could be used in generation of optical bottle beams^54^ and trapping microparticles^55^. In nonlinear media, abruptly autofocusing spatiotemporal beams are able to reshape into nonlinear intense light-bullet wavepackets propagating over extended distances^56^.

We assume that the two-dimensional abruptly autofocusing Airy beam carrying an optical vortex characterized by the topological charge *m* is in the following form^57^

2 $$\psi \,(x,y,z=0)=A\cdot Ai\,[\pm (\frac{{r}_{0}-r}{w})]\,\exp \,[\pm a(\frac{{r}_{0}-r}{w})]\times {(x+iy)}^{m},$$

where *A* is the amplitude of the abruptly autofocusing Airy beam, *w* = 0.5 is the beam width, *r*_0_ is a constant which determines the radial position of the main Airy ring^57^, ± represent the abruptly autofocusing Airy beam self-accelerate towards inward and outward, respectively, and *a* = 0.05 is the decay factor^1^.

**Figure 6:**
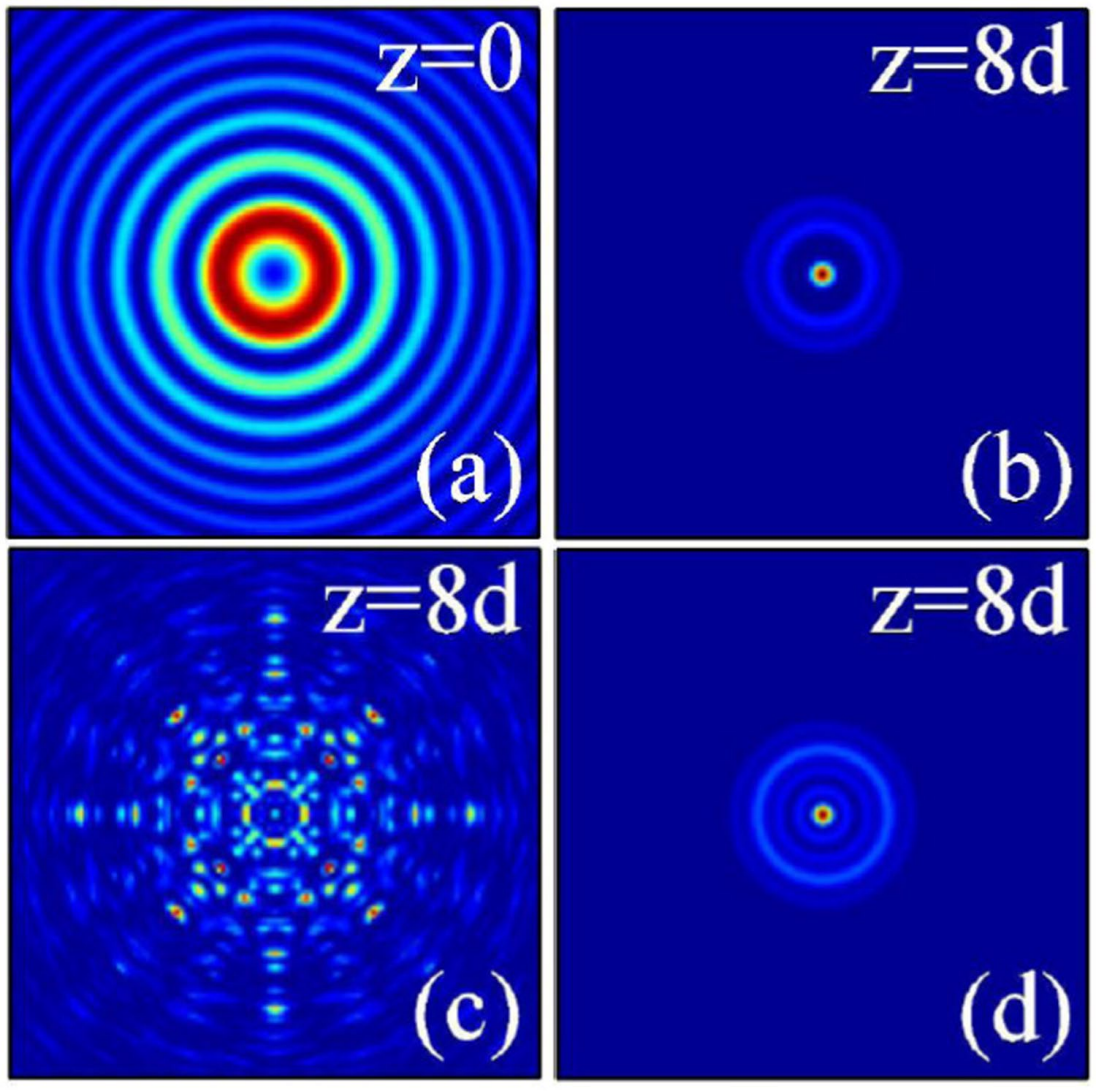
The intensity distributions of inward accelerating abruptly autofocusing Airy beam at different propagation distances in (**a**–**c**) local (*σ* = 0), and (**d**) nonlocal nonlinear media, respectively. The initial parameters are *m* = 0, *r*_0_ = 0.5, *A* = 1 (**a**,**b**), and *A* = 12 (**c**,**d**). The degree of nonlocality of Fig. 6(d) is *σ* = 5.

In Fig. (6), we show the dynamics of two-dimensional inward accelerating abruptly autofocusing Airy beam propagating in local [Fig. 6(a–c)] and nonlocal [Fig. 6(d)] nonlinear media with the topological charge *m* = 0. When the amplitude of the beam is smaller, it is clear that the beam abruptly autofocuses after a certain distance of propagation, as shown in Fig. 6(b). Interestingly, the beam profile asymptotically takes the form of a Bessel beam^55^, i.e., a first order nondiffracting Bessel field [Fig. 6(b)]. Because of the beam accelerates inward, the profile of the Airy beam become smaller after autofocusing. When the amplitude of the beam is larger (strong self-focusing effect), symmetric breaking of the beam will happen due to the collapse effect, as shown in Fig. 6(c). We can also obtain stationary propagation and nonlinear autofocusing of such circularly symmetry Airy beam in nonlocal nonlinear media [Fig. 6(d)].

**Figure 7:**
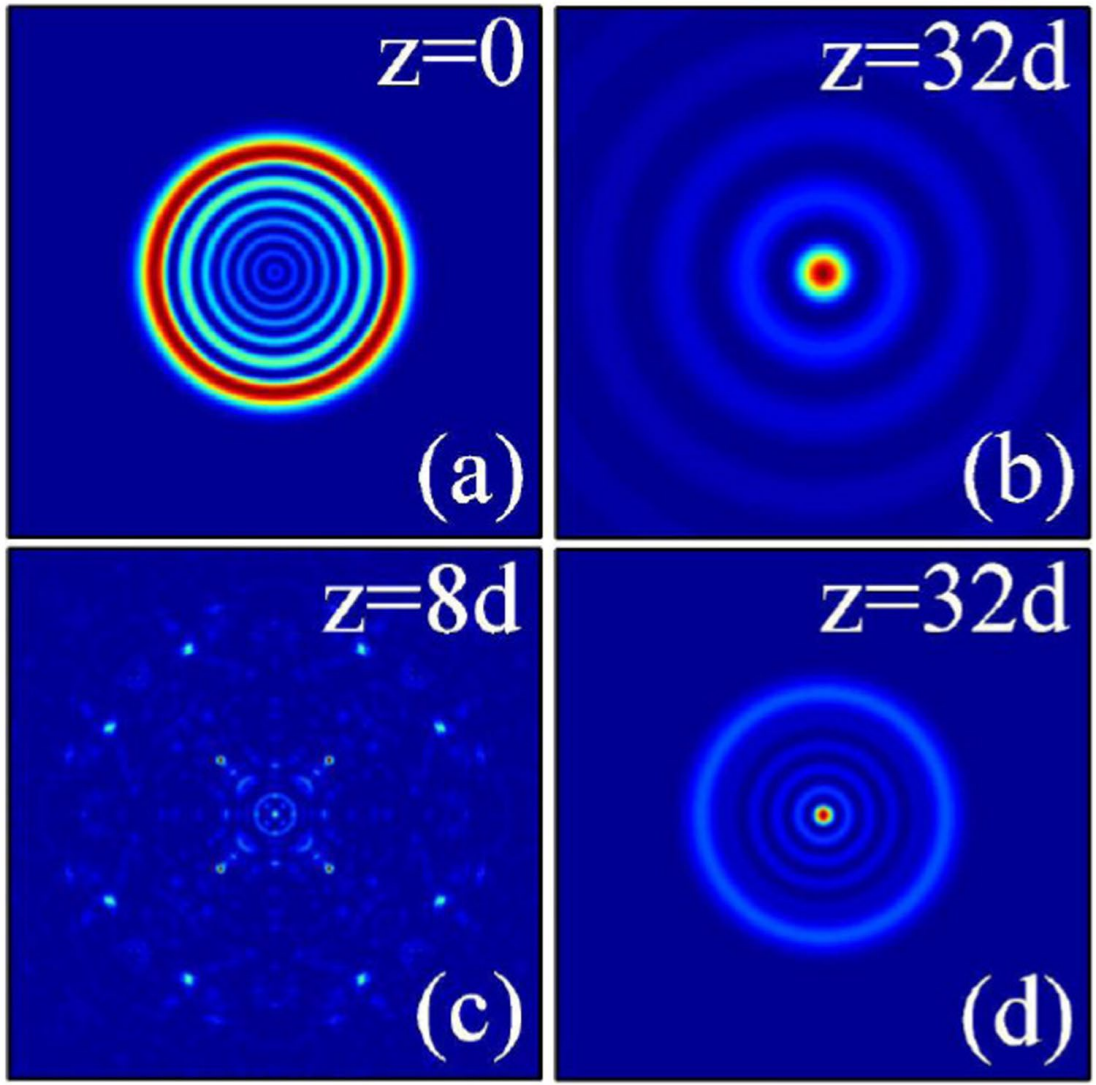
The intensity distributions of outward accelerating abruptly autofocusing Airy beam at different propagation distances in (**a**–**c**) local (*σ* = 0), and (**d**) nonlocal nonlinear media, respectively. The initial parameters are *m* = 0, *r*_0_ = 5, *A* = 1 (**a**,**b**), and *A* = 12 (**c**,**d**). The degree of nonlocality of Fig. 7(d) is *σ* = 5.

Similarly, we show in Fig. (7) the dynamics of two-dimensional outward accelerating abruptly autofocusing Airy beam propagating in local [Fig. 7(a–c)] and nonlocal [Fig. 7(d)] nonlinear media with the topological charge *m* = 0. When the amplitude of the beam is smaller, the beam will also abruptly autofocuses after a certain distance of propagation and takes the form of a Bessel beam^55^, as shown in Fig. 7(b). Whereas, because of the beam accelerates outward, the beam always expand during propagation, and the beam size of the Airy beam will be larger than its initial size after autofocusing. We can also obtain the collapse phenomena and the stationary propagation of the beam in local [Fig. 7(c)] and nonlocal [Fig. 7(d)] nonlinear media when the amplitude of the beam is larger enough, respectively. With the help of the attractive force induced by the nonlocality, from Fig. 7(b,d), we can see clearly the profile (radius) of the beam becomes smaller.

**Figure 8:**
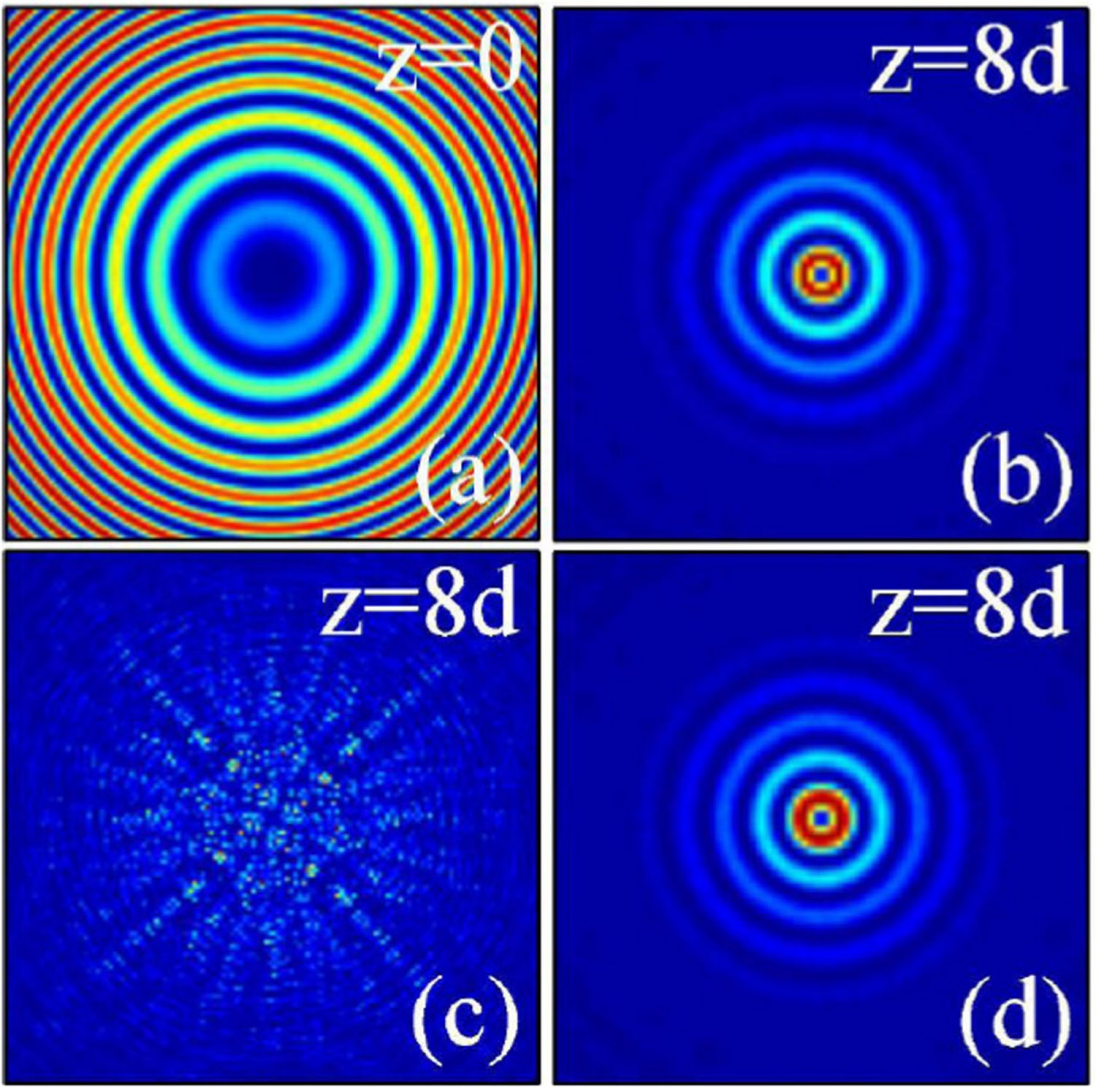
The intensity distributions of inward accelerating abruptly autofocusing Airy beam at different propagation distances in (**a**–**c**) local (*σ* = 0), and (**d**) nonlocal nonlinear media, respectively. The initial parameters are *m* = 1, *r*_0_ = 0.5, *A* = 0.5 (**a**,**b**), and *A* = 3 (**c**,**d**). The degree of nonlocality of Fig. 8(d) is *σ* = 5.

**Figure 9:**
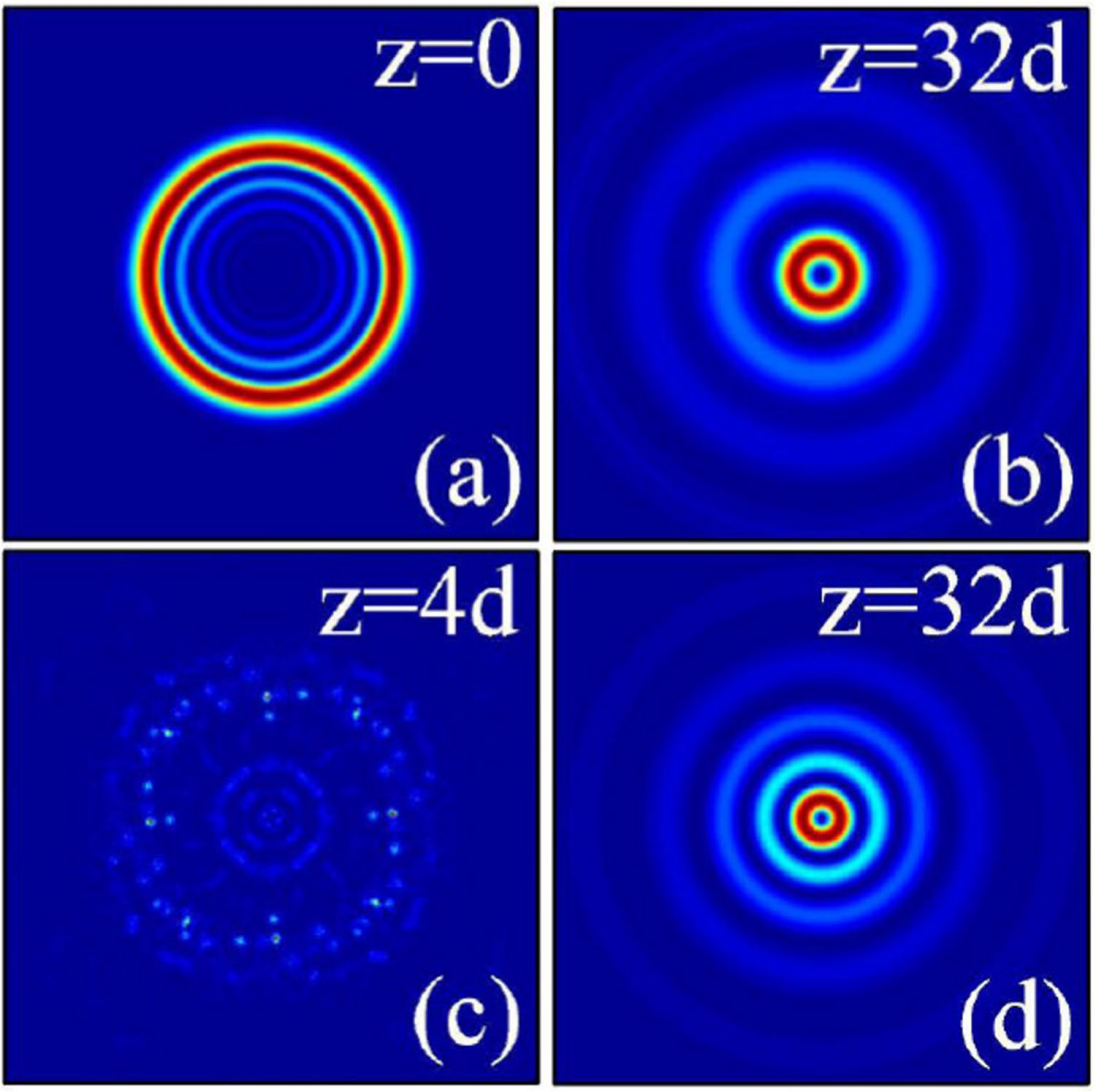
The intensity distributions of outward accelerating abruptly autofocusing Airy beam at different propagation distances in (**a**–**c**) local (*σ* = 0), and (**d**) nonlocal nonlinear media, respectively. The initial parameters are *m* = 1, *r*_0_ = 5, *A* = 0.5 (**a**,**b**), and *A* = 2 (**c**,**d**). The degree of nonlocality of Fig. 9(d) is *σ* = 5.

When *m* ≠ 0, Eq. (8) represents an abruptly autofocusing Airy beam carrying an optical vortex with the topological charge *m*. In Figs (8 and 9), we show the intensity distributions of the inward and outward accelerating abruptly autofocusing vortex Airy beam with the topological charge *m* = 1 at some propagation distances in nonlinear media [(a–c) local and (d) nonlocal], respectively. From Figs 8(b) and 9(b), we can conclude that these Airy beams will also abruptly autofocuses after some propagation distance, which are similar with the case *m* = 0. The different results are that there always exist an optical vortex in the beam center after their autofocusing, on the contrary, no vortex exists when the topological charge is *m* = 0. When *m* ≠ 0, the abruptly autofocusing beam carrying angular momentum, which should be conservative during propagation^27^. Thus the beams take the form of the Laguerre Gaussian beam with zero intensity distributions in the center^57^. Their intensity peaks locate at the first ring of the beam.

With increasing the amplitude of the Airy beam, collapse of the self-accelerating beams in local nonlinear media will happen again, as shown in Figs 8(c) and 9(c). The Airy beams with higher order topological charge are found to be more easier to collapse for that they have a worse stability than that with lower order topological charge. In nonlocal media, nonlocality provides a long-range attractive force on the autofocusing Airy beams carrying angular momentum, leading to the stationary propagation and autofocusing [Figs 8(d) and 9(d)] which always collapse in local media.

## Discussions

The properties of Airy beams have drawn much attention during the past decade. Several works have concentrated on the propagation dynamics of the Airy beam, such as ballistic motion and self-healing^49^. As to the self-healing properties of an Airy beam carrying angular momentum, seldom people noticed the effect of the topological charge and the location of the vortex^50, 51^. In nonlinear media, the beams can propagate stably when the linear diffraction and nonlinear self-focusing balance each other. If the strength of self-focusing is stronger enough than the strength of linear diffraction, the Airy beams will suffer from catastrophic collapse^24–27^.

In local nonlinear media, two-dimensional fundamental Airy beams with higher powers always collapse into particles induced by strong self-focusing effect^24, 25^. When a vortex Airy beam propagating in a nonlinear media, the locations of collapse can be controlled by the initial power, vortex order, and modulation parameters^26^. However, no one knows how to prevent the collapse of the Airy beams. In this paper, we also show in Figs 2(c) and 5(a) the collapse of two-dimensional fundamental and vortex Airy beams in local nonlinear media. However, the collapse of Airy beams can be prevented in nonlocal media, as shown in Figs 2(d) and 5(b). The effect of nonlocality is also applicable to two-dimensional abruptly autofocusing Airy beams [Figs (6–9)] which has not been studied before.

In summary, we have investigated numerically dynamics and collapse of two-dimensional Airy beam in nonlocal nonlinear media. The stability and self-healing properties of the beams depended crucially on the location and topological charge of the vortex when the beam carry angular momentum. We also demonstrated the propagation of abruptly autofocusing Airy beam in detail. In the regime of strong self-focusing, we obtained the stationary propagation of two-dimensional Airy beams with the help of nonlocality as long as it is symmetric and has a positive definite Fourier spectrum, which always collapse in local nonlinear media.

It should be emphasized that our results obtained here are not only applicable to two-dimensional conventional and abruptly autofocusing Airy beams, but also applicable to other two-dimensional self-accelerating beams. In the future work, we would like to study the dynamics of three dimensional Airy beams^1, 27, 56, 58, 59^ with large power in nonlocal media. We will show whether nonlocality could suppress their catastrophic collapse, and this will be interesting of course.

## Methods

### Basic equations

In this paper, we consider a two-dimensional Airy beam propagating in a nonlocal nonlinear media. The slowly varying beam envelope of the Airy beam *ψ* (*x*, *y*, *z*) can be described by the normalized nonlocal nonlinear Schrödinger equation^42^,

3 $$i\frac{\partial \psi }{\partial z}+\frac{{\partial }^{2}\psi }{\partial {x}^{2}}+\frac{{\partial }^{2}\psi }{\partial {y}^{2}}+\delta n(I)\psi =0,$$

where the variables *x*, *y* and *z* are the normalized transverse and longitudinal coordinates, respectively, scaled by the characteristic transverse width *w* and the corresponding Rayleigh range *d* = *kw* ^2^/2^22^. *δn*(*I*) is the nonlinear refractive index change of the nonlocal nonlinear media, which can be represented by the following convolutional form:

4 $$\delta n(I)=\int R(\overrightarrow{r}-\overrightarrow{r}^{\prime} ){|\psi (\overrightarrow{r}^{\prime} ,z)|}^{2}d\overrightarrow{r}^{\prime} ,$$

here $\overrightarrow{r}(x,y)$ is the vector spatial coordinate, $R(\overrightarrow{r})$ is the normalized nonlocal response functions of the media which satisfies the normalized condition $\int R(\overrightarrow{r})d\overrightarrow{r}=1$ ^44^.

The system of Eq. (1) conserves the total power

5 $$P=\int \int {|\psi (x,y)|}^{2}dxdy,$$

and the Hamiltonian

6 $$H=\int ({|\nabla \psi |}^{2}-\frac{1}{2}{|\psi |}^{2}\delta n(I))d\overrightarrow{r},$$

respectively.

### Nonlocal response function and Fourier transform

Without loss of generality, in this paper, we consider the case of the so-called Gaussian nonlocal response functions^42, 60^

7 $$R(\overrightarrow{r}-\overrightarrow{r}^{\prime} )=\frac{1}{\pi {\sigma }^{2}}\,\exp \,[-\frac{{(\overrightarrow{r}-\overrightarrow{r}^{\prime} )}^{2}}{{\sigma }^{2}}],$$

with a characteristic width *σ* which represents the degree of the nonlocality, as displayed in Fig. 1(a). We also show in Fig. 1(b) the Fourier transform $\tilde{R}(\overrightarrow{k})$ of $R(\overrightarrow{r})$ by the following calculation^36^

8 $$\tilde{R}(\overrightarrow{k})=\int R(\overrightarrow{r})\exp (i\overrightarrow{k}\cdot \overrightarrow{r})d\overrightarrow{r}=\exp \,[-\frac{{\sigma }^{2}{k}^{2}}{4}],$$

with $\overrightarrow{k}={k}_{x}\overrightarrow{x}+{k}_{y}\overrightarrow{y}$ is the wave vector in frequency domain.
